# Mitigating Ni and Cu ecotoxicity in the ecological restoration material and ornamental *Primula forbesii* Franch. with exogenous 24-epibrassinolide and melatonin

**DOI:** 10.1038/s41598-024-67093-8

**Published:** 2024-07-11

**Authors:** Hongchen Yang, Jian Zhao, Xiancai Yin, Keying Ding, Xinhui Gao, Yuxin Cai, Yuanzhi Pan, Beibei Jiang, Qinglin Liu, Yin Jia

**Affiliations:** College of Landscape Architecture, Sichuan Agricultural University, Chengdu, 611130 China

**Keywords:** *Primula*, Exogenous phytohormone, Gas exchange parameters, Lipid peroxidation, Antioxidant enzymes, AsA-GSH, Plant physiology, Plant stress responses

## Abstract

Nickel (Ni) and copper (Cu) contamination have become major threats to plant survival worldwide. 24-epibrassinolide (24-EBR) and melatonin (MT) have emerged as valuable treatments to alleviate heavy metal-induced phytotoxicity. However, plants have not fully demonstrated the potential mechanisms by which these two hormones act under Ni and Cu stress. Herein, this study investigated the impact of individual and combined application of 24-EBR and MT on the growth and physiological traits of *Primula forbesii* Franch. subjected to stress (200 μmol L^–1^ Ni and Cu). The experiments compared the effects of different mitigation treatments on heavy metal (HM) stress and the scientific basis and practical reference for using these exogenous substances to improve HM resistance of *P. forbesii* in polluted environments. Nickel and Cu stress significantly hindered leaf photosynthesis and nutrient uptake, reducing plant growth and gas exchange. However, 24-EBR, MT, and 24-EBR + MT treatments alleviated the growth inhibition caused by Ni and Cu stress, improved the growth indexes of *P. forbesii*, and increased the gas exchange parameters. Exogenous MT effectively alleviated Ni stress, and 24-EBR + MT significantly alleviated the toxic effects of Cu stress. Unlike HM stress, MT and 24-EBR + MT activated the antioxidant enzyme activity (by increasing superoxide dismutase (SOD), peroxidase (POD), and catalase (CAT)), significantly reduced reactive oxygen species (ROS) accumulation, and regulated ascorbate and glutathione cycle (AsA-GSH) efficiency. Besides, the treatments enhanced the ability of *P*. *forbesii* to accumulate HMs, shielding plants from harm. These findings conclusively illustrate the capability of 24-EBR and MT to significantly bolster the tolerance of *P. forbesii* to Ni and Cu stress.

## Introduction

The growing human population, rapid industrialization and urbanization, and intensive farming practices have all contributed to the large-scale discharge of hazardous metals into ecosystems^1^. These contaminants continuously accumulate in ecosystems, causing significant soil degradation and detrimental effects on plants^2,3^. Heavy metal contamination of soil is of great concern owing to its high toxicity, resistance to degradation, and high enrichment^4^. Excess essential and non-essential HMs, including mercury (Hg), chromium (Cr), nickel (Ni), copper (Cu), and zinc (Zn), can be toxic to plants and, in severe cases, cause plant death^5^. The China Soil Pollution Survey Bulletin (https://www.mee.gov.cn/) showed that local Ni and Cu levels exceed the national limits by 4.80 and 2.10%, respectively, ranking second and fourth among inorganic pollutants. These two elements are the main pollutants of China’s arable land.

Nickel and Cu are indispensable trace elements in the growth and development of higher plants. The beneficial Ni concentration range differs among plant species. Exceeding this range causes plant nutritional and physiological disorders^6^. High Ni, Cu, and Cr contents in the soil will cause plants to absorb them excessively, hindering the absorption and transport of other nutrient elements. Thus, excess Ni, Cu, and Cr destroys physiological, biochemical, and metabolic functions^7,8^, reducing plant growth, development, and yield^9^. Excessive Ni disrupts phytohormone homeostasis, inhibiting plant growth and development. This inhibition is characterized by phenotypic changes in the plant, including abnormal leaf color, impaired root growth, and changes in enzyme activities^10^. Excessive Cu uptake can cause various problems, including reduced seed germination, stunted root and stem growth, leaf chlorosis, and mineral nutrient deficiencies^11^. Additionally, HM toxicity disrupts ROS balance, activates lipid membrane oxidation, and enhances the activities of corresponding MDA and various antioxidant enzymes. Therefore, plants have evolved antioxidant defense mechanisms to prevent excessive ROS production. In the antioxidant defense system, the AsA-GSH cycle is important in ROS detoxification and maintaining cellular oxidation–reduction homeostat^12^. To some extent, plants can regulate HM stress, but plant defenses are overwhelmed as the degree of HM pollution increases. In order to mitigate phytotoxicity, it is imperative to minimize plant damage efficiently and sustainably. Growth hormones regulate response to adverse conditions in plants. Therefore, understanding their functional mechanisms is crucial for enhancing plant resistance to various stresses. Studies have demonstrated that 24-EBR and MT can modulate enzyme activity and mitigate oxidative damage^11^. Thus, exogenous hormones are applied to alleviate Cu toxicity in plants and mitigate other biotic stresses.

Brassinosteroids (BRs) are a class of steroid hormones widely found in plants. Moreover, BRs are known for promoting plant growth and development and ameliorating the negative consequences of abiotic stresses such as drought, salinity, and HM toxicity^13,14^. Brassinosteroids remove ROS by binding them (ROS) to membrane proteins, preventing plants from absorbing HMs thus stabilizing the electrical properties and enzyme activity of cell membranes^15^. For example, 24-EBR treatment can reduce the accumulation of HMs in plants, thus mitigating the toxic effects. Simultaneously, 24-EBR can promote plant growth and development, increase chlorophyll content and photosynthetic efficiency, and augment biomass accumulation. Additionally, the 24-EBR application induced the production of antioxidant enzymes, such as superoxide dismutase (SOD) and peroxidase (POD), to scavenge ROS. This process has also been found to induce the production of proline and soluble proteins to maintain the osmotic potential of cells^16^. Copper stress and 24-EBR treatment enhanced the morphological parameters of grape roots (*Vitis vinifera* L). Simultaneously, 24-EBR significantly increased the activities of SOD, POD, and CAT in the aboveground and belowground plant parts. Application of 24-EBR increased levels of ascorbate peroxidase (APX), monodehydroascorbate reductase (MDHAR), glutathione reductase (GR), and dehydroascorbate reductase (DHAR) by regulating the AsA-GSH cycle balance^11^. Further, 24-EBR improved plant growth under Cu stress and reduced ROS production by promoting antioxidant and osmolyte accumulation^6^. The 24-EBR hormone also plays a critical role in mitigating the toxic effects of Ni stress and osmoregulation in plants^17^. Kanwar et al.^18^ showed that 24-EBR improves the morphological and physiological properties of plants by regulating the activity of various antioxidants, thus attenuating the toxic effects of Ni.

The natural signaling molecule *N*-acetyl-5-methoxytryptamine (MT), widely found among plants and animals, has powerful antioxidant properties that can effectively protect plants from the HMs in the soil^19^. Besides, MT is an ecologically safe molecule with high feasibility and low-cost characteristics for environmental application, significantly improving the tolerance of various plant species to environmental pollution^20^. This plant hormone, MT, affects basic physiological functions, including plant growth and development and photosynthesis, and enhances plant tolerance to drought^21^, high temperature^22^, salinity^23^, and other abiotic stresses. Advanced detection technologies have identified MT in numerous plant species. Moreover, it has been repeatedly shown that MT significantly alleviates HM stress^24–27^. For example, MT mitigates Cu^2+^ toxicity by improving Cu^2+^ carbon fixation, carbon metabolism, and scavenging ROS in cucumber (*Cucumis sativu*s L.). Exogenous MT markedly suppressed the dwarf phenotypes and plant fresh weight reduction caused by excess Cu^2+^. Furthermore, transcriptome analysis indicated that MT induced an effective defense against excess Cu^2+^ by inhibiting ROS production and enhancing the transcript and enzymatic activity of key antioxidant system components^28^. Similarly, Jahan et al.^29^ showed that MT enhanced tomato (*Solanum lycopersicum* L.) growth characteristics, increased photosynthetic efficiency, and up-regulated chlorophyll synthesis genes. Meanwhile, MT suppressed ROS production, improved the antioxidant defense system, dramatically reduced Ni accumulation, and improved the mineral-nutrient homeostasis of plants.

*Primula forbesii* Franch. is endemic to China and mainly distributed in Sichuan and Yunnan Provinces^30^. *P. forbesii* blooms in winter and spring and has a high ornamental value and pleasant aromatic odor, making it very valuable for gardening. However, few studies have discussed the physiology of *P. forbesii*, except for the effects of drought, heat, and cold resistance. The effect of HM stress on *P*. *forbesii* remains unknown. Nonetheless, recent studies have shown that *P. forbesii* seedlings tolerate Cd stress, but low Cd stress has negligible effects on *P. forbesii*. However, high Cd concentrations are toxic against normal *P. forbesii* growth. Exogenous salicylic acid (SA) significantly reduced the toxic effects of Cd stress on *P. forbesii* seedlings and decreased Cd accumulation in plants^30,31^. Furthermore, external 24-EBR successfully reduced Cd stress and enhanced the ability of *P. forbesii* to withstand Cd stress^32^. Thus, hydroponics have been extensively employed to investigate HM toxicity in plants because this method precisely determines the physiological parameters and metabolic indexes^33^. Like exogenous 24-EBR, MT also protects plants from HM toxicity. However, the effects of the phytohormone on *P. forbesii* under HM stress are limited. Thus, this study examined the effects of these two hormones on Ni- and Cu-stressed *P. forbesii* by systematically treating *P. forbesii* with exogenous 24-EBR and MT. Differences in growth, photosynthetic activity, antioxidant levels, and Ni and Cu contents of the plants were analyzed. The results provide a new perspective for a deeper understanding of the effects of plant hormones on HM tolerance.

## Materials and methods

### Experimental materials

Seeds of ‘Pink violet’, a new variety independently cultivated by Dr. Yin Jia of the Sichuan Agricultural University and with independent intellectual property rights, were used for this study. Live *P. forbesii* seedlings were obtained by sowing seeds in the greenhouse of the College of Landscape Architecture, Sichuan Agricultural University (103° 51′ 44″ E, 30° 42′ 17″ N). The seeds were harvested from naturally pollinated *P. forbesii* in the greenhouse in the same year, shade-dried, cold-treated at 4 °C, and sown in May and June of the same year. Seedlings exhibiting uniform growth and good health were selected at the five to six true leaves stage. The selected seedlings were transferred to indoor light culture racks for acclimatization before experimental treatments in July and August.

### Preparation of 1/8-strength modified Hoagland’s nutrient, Ni, and Cu solutions

Table 1 contains the composition of the Hoagland nutrient solution used in this study. NiCl_2_·2H_2_O and CuSO_4_·5H_2_O were the raw materials for Cu and Ni stock solutions. The stock solutions were filtered through 0.45-μm nylon membrane filters to remove impurities.

**Table 1:** Modified Hoagland hydroponic nutrient solution formula.

Compound	Standard concentration	1/8 times concentration
Ca(NO_3_)_2_·4H_2_O	1180.75	147.59
KNO_3_	505.55	63.19
KH_2_PO_4_	136.09	17.01
MgSO_4_·7H_2_O	49.29	6.16
MnSO_4_·4H_2_O	0.32	0.04
Na_2_MoO_4_·2H_2_O	0.01	0.01
ZnSO_4_·7H_2_O	0.23	0.03
CuSO_4_·5H_2_O	0.07	0.01
FeSO_4_·7H_2_O	5.56	0.69
EDTA-Na_2_	74.85	9.36
H_3_BO_3_	27.82	3.48

### Growth conditions

Plants were grown on culture racks with controlled environmental conditions, day/night temperatures of 25–20 °C, light/dark periods of 16/8 h, relative humidity of approximately 75.00%, and a light intensity of 4000 lx.

### Experimental design

Table 2 shows the experimental design of this study. The experiment was a hydroponics system using *P. forbesii* seedlings of uniform height and growth. The plants were grouped into orchid vials containing 500 mL of 1/8-strength modified Hoagland’s nutrient solution (pH = 5.8 ± 0.2). The experiment was conducted with 12 plants each, three biological replicates, over three weeks. Nickel and Cu (both at a concentration of 200 μmol L^–1^) stress and foliar application of the exogenous hormones 24-EBR (0.1 μmol L^–1^) and MT (100 μmol L^–1^) were selected based on a preliminary experiment. The treatments were: (1) a blank control (using 1/8-strength modified Hoagland’s solution) treated for two weeks. (2) 24-EBR treatment applied by spraying onto the adaxial and abaxial surfaces of the leaves to mitigate Ni and Cu contamination. (3) MT treatments applied by spraying onto the adaxial and abaxial surfaces of the leaves to mitigate Ni and Cu contamination; (4) combined 24-EBR and MT treatments applied by spraying onto the adaxial and abaxial surfaces of the leaves to mitigate Ni and Cu contamination.

**Table 2:** Experimental design.

	Treatments	Concentration
CK	1/8 hoagland hydroponic	
Ni	Ni	Ni 200 μmol L^–1^
	24-EBR + Ni	24-EBR 0.1 μmol L^–1^
	MT + Ni	MT 100 μmol L^–1^
	24-EBR + MT + Ni	
Cu	Cu	Cu 200 μmol L^–1^
	24-EBR + Cu	24-EBR 0.1 μmol L^–1^
	MT + Cu	MT 100 μmol L^–1^
	24-EBR + MT + Cu	

Three weeks after treatment, *P. forbesii* plants were harvested, washed with distilled water, and separated into aboveground and underground parts for subsequent physiological, biochemical, and enzyme analyses. The fresh and dry weights of both aboveground and underground parts were measured using the gravimetric method. The remaining fresh samples were frozen and stored at − 80 °C. For dry weight determination, samples were dried in a vacuum oven at 105 °C for 30 min and then at 80 °C until a constant weight was achieved. Nitric acid digestion of the samples was performed using inductively coupled plasma mass spectrometry (ICP-MS) to assess Ni and Cu accumulation. Simultaneously, other physiological parameters were measured immediately after plant harvest. Approximately eight weeks after treatment, plants began to flower. The days until the first flowering (post-treatment) were recorded for each treatment and the total number of flowers per plant was calculated six weeks after treatment.

### Determination of morphological and growth indicators

Plant height (i.e., vertical distance from the base of the stem to the highest part of the plant), root length (vertical distance from the base of the stem to the longest main root of the plant), and crown width (width of *P. forbesii*, based on an average of long and short edges) were averaged for each treatment. The fresh weights (FW) of aboveground and belowground parts were determined. The weighted parts were dried at 105 °C for 30 min and then at 80 °C until constant weight; the dry weight (DW) of the aboveground and belowground parts was then recorded from these dried materials.

### Measurement of photosynthetic gas exchange parameters

The net photosynthetic rate (Pn), stomatal conductance (Gs), and transpiration rate (Tr) were measured using a LI-6400 portable photosynthesis system (LI-COR Biosciences, NE, USA). The light intensity and CO_2_ concentration were 1000 ± 10 μmol m^-2^ s^−1^ and 400 ± 10 μmol mol^−1^, respectively.

### Determination of ROS content and membrane lipid peroxidation indexes

The MDA contents were determined following the Heath and Packer^34^ method. The contents of superoxide anion (O^2−^) and hydrogen peroxide (H_2_O_2_) in the plant samples were determined following the method of Elstner and Heupel^35^ and Wang et al.^36^, respectively.

### Determination of antioxidant system content

The total antioxidant capacity (TAC) (mmol g^−1^ FW) was assessed using the FRAP method with the S0116 total antioxidant capacity assay kit (Beyotime, Shanghai, China). Next, SOD activity was determined following the method of Hess and Foster^37^, and CAT activity was measured spectrophotometrically following the Aebi^38^ procedure. The POD activity was determined using the method of Shah et al.^39^. Finally, the activities of SOD, POD, and CAT were expressed as units (U) per gram of fresh weight (FW), with one enzyme activity unit (U) defined as the reduction of light absorption value by 0.1 per minute.

### AsA-GSH cycle-related enzyme activity assay

The activity of APX (expressed as μmol g^−1^ min^−1^ FW) was determined at 290 nm following extraction with 2 mM ascorbic acid^40^. Next, AsA (μg g^−1^ FW) and GSH (μmol g^−1^ FW) were measured according to the methods outlined by Law et al.^41^ and Aravind and Prasad^42^, respectively. DHAR, MDHAR, and GR were determined according to Jia et al.^32^.

### Determination of Ni and Cu contents

Nickel and Cu concentrations in the aboveground and belowground biomass were analyzed using Agilent 7700 ICP-MS (Agilent Technologies, CA, USA). Specific methods were determined according to Jia et al.^32^.

### Statistical analysis

Statistical analysis was conducted using Excel (Microsoft Corp., Redmond, WA, USA) and SPSS Statistics 27.0 (IBM Corp., Armonk, NY, USA). A one-way analysis of variance (ANOVA) was employed to ascertain the significant effects of treatments, followed by Duncan’s multiple-range test for mean comparisons. In all figures, distinct letters indicate significant differences (*p* < 0.05). Graphical representations were generated using Origin 2021 (OriginLab, MA, USA).

## Results and discussion

### Effects of 24-EBR and MT on growth indexes of *P. forbesii* under Ni and Cu stress

Table 3 demonstrates the significant negative impacts of 24-EBR and MT on the growth of *P. forbesii* under Ni and Cu single stresses, including plant height and stem length reductions, which seriously affected normal plant growth and development. Exogenous application of the mitigating substances showed some restorative effects, alleviated the damage inflicted by HM stress, and fostered normal plant growth and development. Under Ni stress, exogenous 24-EBR and MT significantly improved the growth parameters of *P. forbesii*. Further, MT addition was particularly effective in restoring the growth parameters of *P. forbesii* to levels approximate to the control, indicating the potential efficacy of MT in ameliorating the adverse effects of Ni stress on plant growth and biomass. Under Cu stress, the exogenous mitigants promoted the *P*. *forbesii* growth, and the combined treatment (MT + 24-EBR) showed the best mitigating effect, resulting in similar growth indexes with the control. Compared with plants under Cu stress alone, the crown width, plant height, aboveground fresh weight, and underground fresh weight of the combined treatment group increased by 67.43%, 65.11%, 41.67%, and 91.10%, respectively, exceeding the corresponding values of the other treatment groups. The growth parameters of *P. forbesii* were much greater than those of the control.

**Table 3:** Effect of 24-epibrassinolide (24-EBR), melatonin (MT), and their combined effect on the growth of *Primula forbesii* under Ni and Cu stress.

Parameter	Crowd width (cm)	Shoot length (cm)	Shoot fresh weight (g)	Shoot dry weight (g)	Root length (cm)	Root fresh weight (g)
CK	11.10 ± 0.20^a^	8.10 ± 0.46^a^	2.85 ± 0.23^b^	0.28 ± 0.06^c^	12.73 ± 0.45^a^	0.55 ± 0.01^a^
Ni	7.77 ± 1.32^b^	6.13 ± 0.86^c^	1.26 ± 0.32^c^	0.22 ± 0.05^c^	8.50 ± 0.95^c^	0.21 ± 0.03^c^
EBR + Ni	10.50 ± 0.10^a^	7.53 ± 0.31^ab^	2.51 ± 0.05^b^	0.42 ± 0.02^b^	11.37 ± 0.74^ab^	0.37 ± 0.06^b^
MT + Ni	11.30 ± 0.62^a^	8.43 ± 0.29^a^	3.38 ± 0.42^a^	0.54 ± 0.04^a^	12.17 ± 1.63^ab^	0.52 ± 0.06^a^
EBR + MT + Ni	8.23 ± 0.42^b^	7.13 ± 0.38^b^	2.55 ± 0.28^b^	0.40 ± 0.02^b^	10.60 ± 0.26^b^	0.51 ± 0.05^a^
CK	11.10 ± 0.20^ab^	8.10 ± 0.46^a^	2.85 ± 0.23^a^	0.28 ± 0.06^b^	12.73 ± 0.45^a^	0.55 ± 0.01^a^
Cu	6.97 ± 1.04^d^	5.13 ± 0.75^c^	1.68 ± 0.08^d^	0.26 ± 0.02^b^	6.63 ± 1.27^c^	0.24 ± 0.05^c^
EBR + Cu	9.80 ± 0.10^bc^	6.53 ± 0.06^b^	2.06 ± 0.08^c^	0.33 ± 0.03^b^	10.07 ± 0.45^b^	0.36 ± 0.01^b^
MT + Cu	8.60 ± 1.18^c^	6.03 ± 0.32^bc^	3.13 ± 0.21^a^	0.30 ± 0.05^b^	8.87 ± 0.70^b^	0.36 ± 0.07^b^
EBR + MT + Cu	11.67 ± 0.38^a^	8.47 ± 1.04^a^	2.38 ± 0.20^b^	0.43 ± 0.03^a^	12.67 ± 0.78^a^	0.48 ± 0.10^a^

Data are expressed as means ± S.D. Values labeled with different lowercase letters are significantly different (*p* < 0.05) according to Duncan’s multiple range test.

CK, control treatment; Ni/Cu, treatment with 200 μmol L^–1^ Ni/Cu; EBR + Ni, 0.1 μmol L^–1^ 24-EBR + 200 μmol L^–1^ Ni; MT + Ni, 100 μmol L^–1^ MT + 200 μmol L^–1^ Ni; EBR + MT + Ni, 0.1 μmol L^–1^ 24-EBR + 100 μmol L^–1^ MT + 200 μmol L^–1^ Ni. EBR + Cu, 0.1 μmol L^–1^ 24-EBR + 200 μmol L^–1^ Cu; MT + Cu, 100 μmol L^–1^ MT + 200 μmol L^–1^ Cu; EBR + MT + Cu, 0.1 μmol L^–1^ 24-EBR + 100 μmol L^–1^ MT + 200 μmol L^–1^ Cu.

Inhibition of plant growth under stress conditions is an important indicator of stress. In this study, Ni and Cu treatments decreased the growth parameters of *P. forbesii*, including crown width, stem, and fresh weights. This result is consistent with previous studies on Cabbage (*Brassica oleracea*)^43^, Sweet potato (*Ipomoea batatas* (L.) Lam.)^44^, and tomato (*Solanum lycopersicum*)^45^. Heavy metal stress hinders plant growth and negatively affects nutrient uptake, metabolism, gas exchange, and cellular osmosis, significantly reducing root, stem, and leaf biomass^46^. For example, Cd stress inhibits the growth of tomato (*Lycopersicon esculentum*) by destroying root morphology^47^. However, it is noteworthy that exogenous application of 24-EBR, MT, and 24-EBR + MT effectively alleviated the inhibition of plant growth. 24-EBR can partially mitigate the effects of HM stress on plant growth by regulating cell division and elongation^48^. Meanwhile, MT, a natural signaling molecule, protects plants from HM stress by regulating several physiological functions^49^. Moreover, applying 24-EBR improved morphological attributes and promoted biomass accumulation in plants under Cu stress^29^. The addition of MT also significantly alleviated the diminished growth parameters of tomato (*Solanum lycopersicum* L.) seedlings under Ni stress, including decreases in stem fresh and dry weights^50^. Thus, the exogenous mitigating substances 24-EBR and MT significantly and positively affect plant growth recovery under HM stress.

### Effects of 24-EBR and MT on photosynthetic gas exchange parameters of *P. forbesii* under Ni and Cu stress

**Figure 1:**
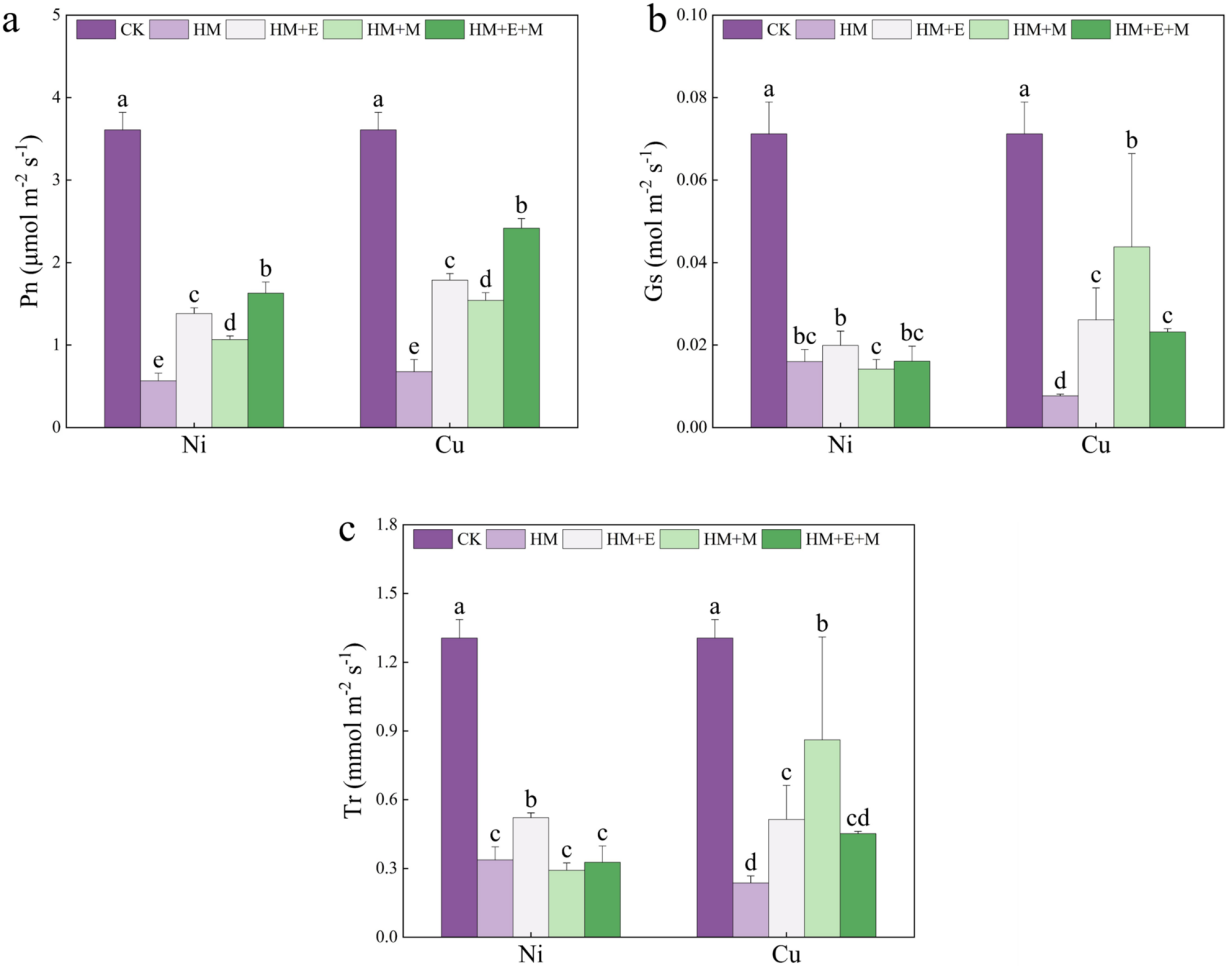
Exogenous hormones effect on the net photosynthetic rate (Pn, **a**), stomatal conductance (Gs, **b**), transpiration rate (Tr,c) of *Primula forbesii* under different treatments. CK, control treatment; HM, treatment with 200 μmol L^–1^ Ni/Cu; HM + E, 200 μmol L^–1^ Ni/Cu + 0.1 μmol L^–1^ 24-EBR; HM + M, 200 μmol L^–1^ Ni/Cu + 100 μmol L^–1^ MT; HM + E + M, 200 μmol L^–1^ Ni/Cu + 0.1 μmol L^–1^ 24-EBR + 100 μmol L^–1^ MT. Data are expressed as means ± S.D. Values labeled with different lowercase letters are significantly different (*p* < 0.05) according to Duncan’s multiple range test.

Figure 1 summarizes the gas exchange parameters recorded during photosynthesis. Nickel and Cu treatments significantly reduced the net photosynthetic rate (Pn), stomatal conductance (Gs), and transpiration rate (Tr) of the plants. Compared with the stressed treatment group, exogenous additions of 24-EBR and MT improved the Pn, Gs, and Tr of the plants. Notably, 24-EBR significantly enhanced these parameters compared with the Ni stress treatment alone, which increased Pn and Tr by 144.43% and 57.00%, respectively. Plants treated with 24-EBR + MT showed no significant difference from those under HM stress alone in either Gs or Tr. However, the combined treatment significantly increases Pn than Ni and Cu stress alone. Compared with Cu stress alone, 24-EBR treatment significantly increased the plant Pn values by 1.64-fold, while MT treatment significantly increased Gs and Tr by 4.69- and 2.64-fold, respectively. Compared with HM stress alone, 24-EBR + MT treatment increased the plant photosynthetic gas exchange parameters, especially Pn, by 2.57-fold.

Heavy metals inhibit photosynthesis and damage photosynthetic organs. Nonetheless, photosynthesis is the key energy for plant growth and development, consistent with this study, where Ni and Cu treatments decreased the Pn, Gs, and Tr values of *P. forbesii*. Previous findings also indicated that Ni stress decreased the Pn, Gs, and Tr contents of tomato (*Solanum lycopersicum* L.) and pepper^29,51^. Application of 24-EBR to *P. forbesii* significantly increased the content of RuBisCo and thus Pn^52^, suggesting that 24-EBR enhances the photosynthetic activity of plants, promoting the growth and development of *P. forbesii*. These results are consistent with the observed growth phenotype of *P. forbesii* after applying 24-EBR to plants under Ni and Cu stresses. Additionally, the MT application protects the photosynthetic system of plants by increasing Pn, Gs, and Tr^51^. Therefore, MT alleviates the weakened photosynthesis and reduces the stomatal closure under HM stress. Altogether, 24-EBR, MT, and 24-EBR + MT exert mitigating effects on the Ni- and Cu-induced reductions in photosynthetic parameters. However, under Ni stress, the effect of 24-EBR was more pronounced, whereas MT application reduced both Gs and Tr. Under Cu stress, 24-EBR + MT and the application of MT alone were superior to the use of 24-EBR alone.

### Effects of 24-EBR and MT on ROS content and lipid peroxidation in *P. forbesii* under Ni and Cu stresses

**Figure 2:**
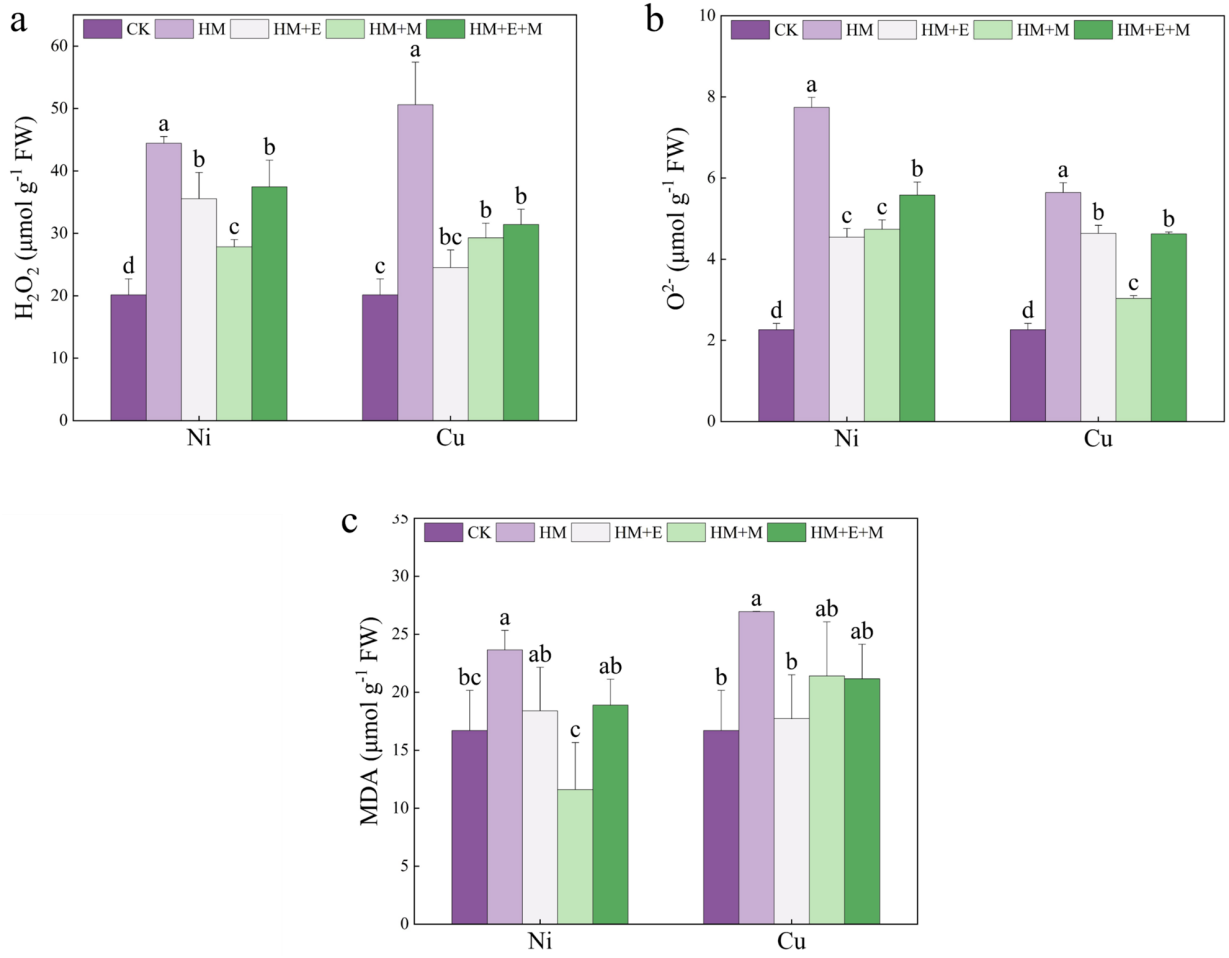
Exogenous hormones effect on the hydrogen peroxide (H_2_O_2_, **a**), superoxide anion (O^2−^, **b**), malondialdehyde (MDA, **c**) of *Primula forbesii* under different treatments. Data are expressed as means ± S.D. Values labeled with different lowercase letters are significantly different (*p* < 0.05) according to Duncan’s multiple range test.

Compared with the control conditions, Ni and Cu stress significantly increased the MDA, H_2_O_2_, and O_2_^−^ contents of *P. forbesii*, indicating that HM stress triggered a substantial accumulation of ROS in plants, leading to cellular damage. Under Ni stress, the MT application was most effective, reducing the MDA content by 52.17%. Under Cu stress, the 24-EBR application reduced the MDA content to the level of the non-stressed treatment. Both HM stresses significantly increased the H_2_O_2_ content of *P. forbesii*. In contrast, MT application to Ni-stressed plants significantly reduced the H_2_O_2_ content. Under Cu stress, the 24-EBR application best mitigated the effect of HM stress. Heavy metal stress significantly elevated the O_2_^-^ content, consistent with recent findings^11^. Under Cu stress specifically, MT application resulted in the most significant decrease in O_2_^-^, showing the greatest O_2_^-^ reducing effect. Under Ni stress, MT application reduced the O_2_^-^ content by 38.82% compared with Ni stress treatment alone. These findings emphasize the potential role of MT and 24-EBR in mitigating the oxidative damage induced by HM stress (Fig. 2).

Heavy metal toxicity in plants exacerbates the excessive accumulation of ROS, the toxic byproduct of metabolic reactions that severely impede plant growth^53,54^. That observation is consistent with the results of this study. *P. forbesii* showed a significant increase in the content of ROS after Ni and Cu stresses. All three treatments decreased O_2_^-^ and H_2_O_2_, with the MT treatment inducing the greatest content decrease for both. However, MT, a multifunctional biomolecule, is important in scavenging ROS across various plant species^55^. Moreover, 24-EBR promotes the antioxidant enzyme defense system to relieve HM stress^56^. Meanwhile, excessive ROS accumulation activates lipid peroxidation and can damage the integrity of cell membranes, impairing cellular functioning^57^. MDA content is a crucial indicator of lipid peroxidation, reflecting the extent of oxidative damage in plants^58^. These experiments also indicated that HM stress increases the MDA content of plants, consistent with the results under Cd^59^ and As stress^60^. The present experiment showed that HM stress increased the MDA content of plants. In contrast, externally applied 24-EBR, MT, and 24-EBR + MT decreased the MDA content, with the most significant effect being MT treatment under Ni stress, which reduced the MDA content to levels below that of the control. Moreover, MT can directly reduce MDA accumulation and protect plant cell membranes from damage^61^. Wu et al.^62^ showed that MT application significantly reduces MDA accumulation in Cd stress environments. Other researchers have shown that 24-EBR can remove excess ROS and reduce MDA production^63^; thus, MT and 24-EBR can alter membrane stability under HM stress^64^. However, MT showed a more significant mitigation effect on alleviating ROS accumulation and lipid peroxidation in *P. forbesii*.

### Effects of 24-EBR and MT on TAC, SOD, POD, and CAT levels of* P. forbesii* under Ni and Cu stress

**Figure 3:**
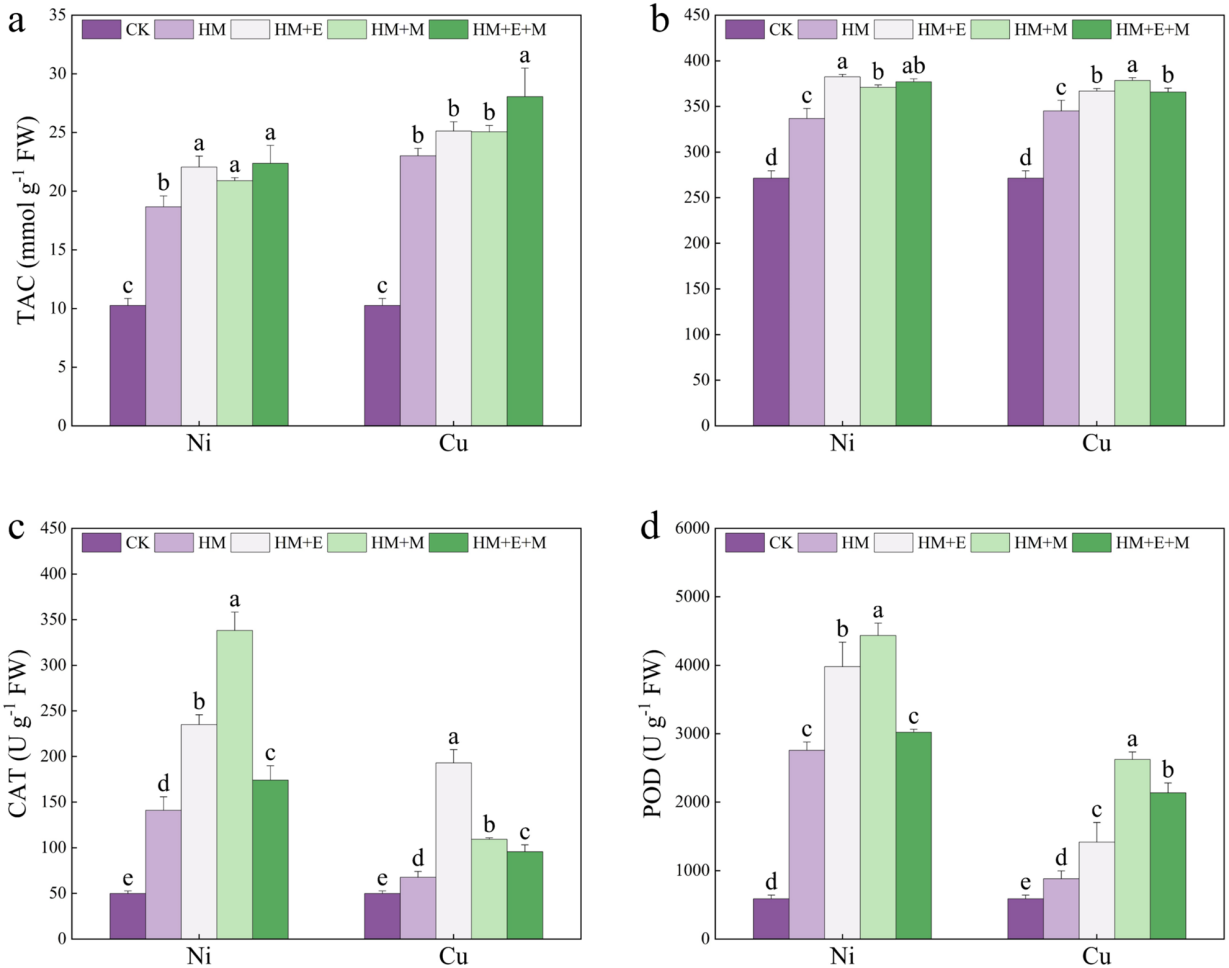
Exogenous hormones effect on the total antioxidant capacity (TAC, **a**), superoxide dismutase (SOD, **b**), peroxidase (POD, **c**), and catalase (CAT, **d**) of *Primula forbesii* under different treatments. Data are expressed as means ± S.D. Values labeled with different lowercase letters are significantly different (*p* < 0.05) according to Duncan’s multiple range test.

The total level of various antioxidant macromolecules, antioxidant macromolecules, and enzymes in a plant, reflecting the TAC of that plant. This parameter can reveal the antioxidant level of the plant and its level of stress to some extent. The TAC content increased under Ni stress, Cu stress, and non-stress treatments. Specifically, TAC increased by 0.82-, 1.15-, 1.04-, and 1.18-fold under Ni stress and by 1.24-, 1.45-, 1.44-, and 1.73-fold under Cu stress, respectively. Under Ni stress, the MT + 24-EBR combination yielded better results. Under Cu stress, the MT + 24-EBR combination enhanced plant TAC by 21.90%, the greatest ameliorative effect observed. Figure 3 shows that HM stress increased SOD, POD, and CAT activity in *P. forbesii*. In contrast, the exogenous mitigating substances also increased the activities of these enzymes under both HM stresses. 24-EBR showed the best performance in promoting *P. forbesii* SOD activity under Ni stress, but the differences among the three exogenous substances were not significant. By comprehensively analyzing the promotion effects, 24-EBR was the best treatment for increasing the SOD activity of plants under Ni stress; under Cu stress, MT was able to maximize the SOD activity. The application of both MT and 24-EBR enhanced the activity of plant POD, with MT having the strongest promotion effect, suggesting that MT has the best promotion effect on the POD activity of *P. forbesii* under Ni stress. All treatments with mitigating substances significantly enhanced POD activity in the Cu stress treatment, and MT had the greatest promotion effect, significantly higher than that of the MT + 24-EBR and 24-EBR, by nearly 2.00-fold than that of Cu stress alone. Nickel stress alone substantially increased the enzyme activity compared with the control group. Exogenous substances enhanced this effect, with the most significant promotion effect observed for MT. Nonetheless, 24-EBR had the greatest promotion effect on Cu-stressed plants, with a 1.85-fold increase compared with Cu stress alone. Melatonin and the MT + 24-EBR combination significantly enhanced CAT activity.

Plants have evolved efficient antioxidant defense systems to overcome the oxidative damage caused by HM stress and mitigate cellular damage. Antioxidant enzymes, including SOD, POD, and CAT, serve as key components. These enzymes catalyze ROS detoxification and positively regulate the antioxidant system, reducing the negative impacts of abiotic stresses on plants and enhancing antioxidant enzyme activity levels^65^. All three mitigating treatments enhanced the activity of antioxidant enzymes and maintained the cellular redox balance^61,66^, consistent with the findings of Zhou et al.^11^. In particular, MT significantly enhanced POD and CAT activities. Melatonin activates the antioxidant enzyme system and regulates enzyme activity, thus reducing the destructive effect of ROS under stress^65^. Wang et al.^67^ showed that under Cd stress, MT improves the growth and development of naked oat seedlings and increases the activity of antioxidant enzymes. These results are supported by the work of Kanwar et al.^18^ and Dalyan et al.^68^, who found that the application of 24-EBR also increased the antioxidant enzyme activity of the plant. Therefore, MT and 24-EBR can regulate the activity of antioxidant enzymes and reduce the adverse effects of HMs on plants.

### Effects of 24-EBR and MT on the AsA-GSH cycle of *P. forbesii* under Ni and Cu stresses

**Figure 4:**
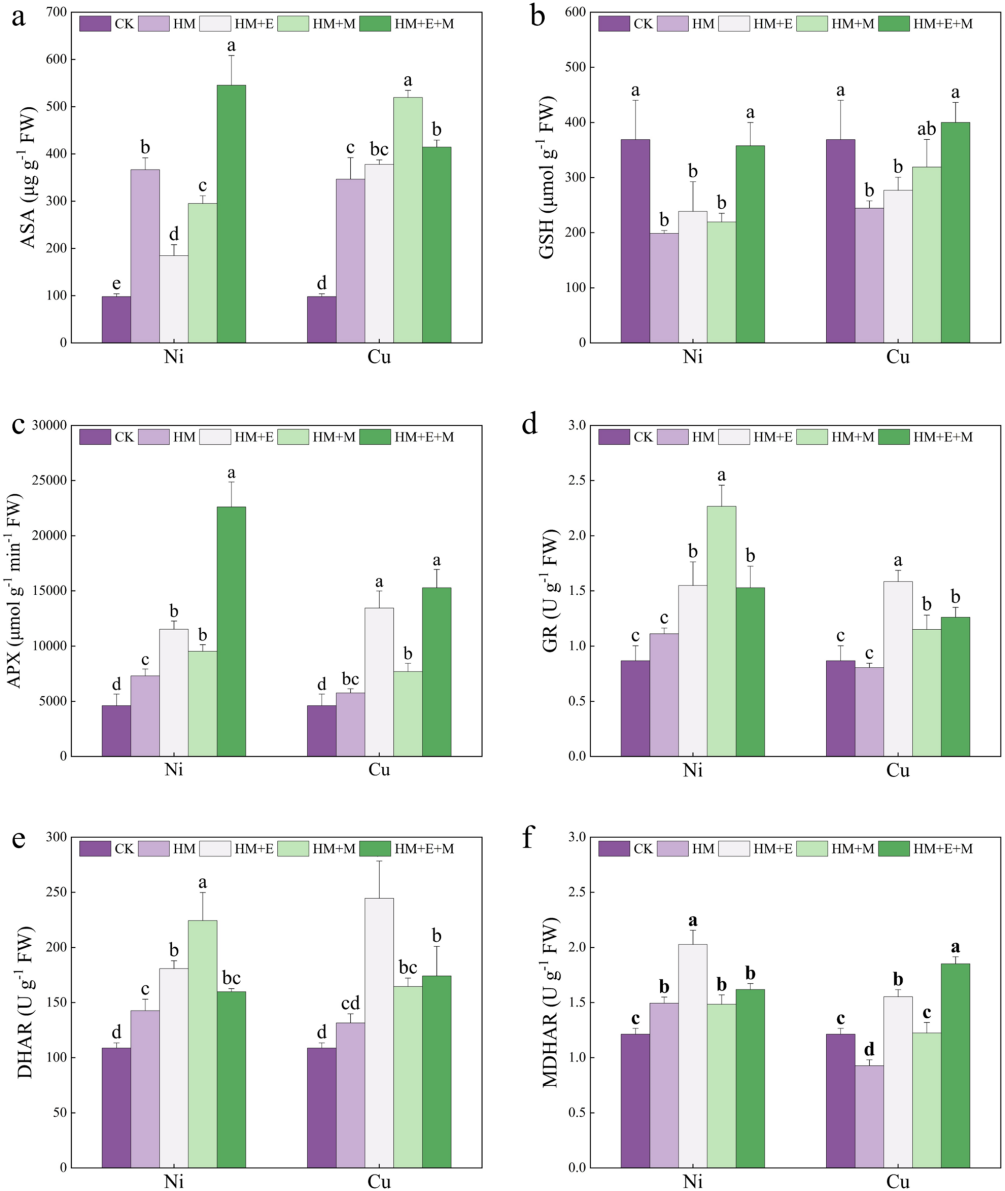
Exogenous hormones effect on the ascorbic acid (ASA, **a**), glutathione (GSH, **b**), ascorbate peroxidase (APX, **c**), glutathione reductase (GR, **d**), dehydroascorbate reductase (DHAR, **e**), and monodehydroascorbate reductase (MDHAR, **f**) of *Primula forbesii* under different treatments. Data are expressed as means ± S.D. Values labeled with different lowercase letters are significantly different (*p* < 0.05) according to Duncan’s multiple range test.

Figure 4 contains the AsA-GSH results. Under Cu stress, all three mitigating regimens increased AsA content to various degrees, and MT had the strongest promoting effect. Under Ni stress, MT and 24-EBR treatments resulted in a significant increase in AsA content compared with the control and a significant decrease compared with the Ni treatment alone. Only MT + 24-EBR caused a significant increase in the AsA content relative to Ni stress alone. *P. forbesii* was stressed by both HMs, and the content of GSH was increased to various degrees by each mitigation regimen. Under Cu stress, the combination of MT + 24-EBR was able to significantly increase the GSH content to levels slightly higher than that of the control. Similar to Cu stress, only the MT + 24-EBR combination significantly increased GSH content under Ni stress, close to the control level. Moreover, HM stress treatments and the mitigating substances increased APX enzyme activity in *P. forbesii* plants compared to the control. In particular, the MT + 24-EBR combination significantly increased APX enzyme activity under Cu stress, especially under Cu stress alone. In contrast, the MT + 24-EBR combination had the strongest promotion effect under Ni stress, increasing APX activity nearly 2.00-fold. The GR enzyme activity decreased slightly under Cu stress than the control and increased slightly under Ni stress. However, Ni stress slightly elevated the GR enzyme activity. Overall, all three mitigation treatments increased the GR activity. Under Cu stress, all mitigation regimens significantly increased GR activity, and 24-EBR was the most effective, significantly more effective than the other mitigation regimens. Under Ni stress, MT promoted GR activity more than the other two regimens. Under single Cu stress, there was no significant increase in DHAR activity compared with the control, but single Ni stress significantly increased DHAR activity. Each mitigation treatment increased DHAR activity to various degrees under all stress conditions. Under Cu stress, each mitigation regimen significantly increased GR activity, and 24-EBR was the most effective, significantly higher than the other mitigation regimens. Under Ni stress, the promotion effect of MT was significantly higher than that of the other two regimens. There was no significant increase in DHAR activity under Cu stress alone compared to the control. Meanwhile, Ni stress alone significantly increased the DHAR activity. Thus, DHAR activity increased to different degrees under each stress. Under Cu stress, there was a significant difference in DHAR activity between the different mitigation treatments, with 24-EBR showing the best results, nearly 86.00% higher than Cu stress alone. Under Ni stress, MT showed the best mitigation effect, significantly greater than the other two mitigating regimens. 24-EBR also mitigated Ni stress to increase DHAR activity, which was significantly higher than that under Ni stress alone. There was a significant difference in the MDHAR activity between the two HM stresses. Under Cu stress alone, MDHAR activity was significantly lower than the control. In contrast, MDHAR activity increased substantially under Ni stress alone compared with the control. Under Cu stress, all three regimens significantly increased the MDHAR activity based on Cu stress alone. The regimens differed significantly, with the MT + 24-EBR combination having the most significant promoting effect. Under Ni stress, the highest MDHAR activity was observed under the 24-EBR treatment. However, there was no significant difference in the MDHAR activity between Ni stress alone and the MT or MT + 24-EBR treatments.

Ascorbate is among the most potent plant cell antioxidants^69^, an electron donor for APX, and reduces H_2_0_2_ to water and is to MDHA. However, MDHA readily disproportionates to DHA or reduces to AsA via MDHAR and regulates plant antioxidant stress^70^. In the context of plant resistance to oxidative stress, GSH is a component of the antioxidant system. Dehydroascorbate is reduced to glutathione disulfide (GSSG), the oxidized form of GSH, and DHAR is involved in this conversion. Furthermore, GR utilizes the NADPH (produced by photosynthesis) generated during photosynthesis to regenerate GSH from the oxidized GSSG^71^. Glutathione can directly eliminate ROS in plants and maintain intracellular REDOX balance. AsA and GSH are involved in the ascorbate–glutathione (AsA-GSH) cycle, serving as crucial antioxidant protective substances that maintain the balance between ROS production and scavenging^72,73^. In agreement with the findings of Jia et al.^32^, the individual Ni and Cu treatments in this study raised AsA but decreased GSH contents, probably because GSH protects plant cells against metal toxicity by acting as a precursor of AsA in the formation of phytochelatins^74,75^. Under stress, *P. forbesii* can maintain redox stability by enhancing the antioxidant system while converting GSH to AsA. The combined 24-EBR + MT application effectively increased GSH content, consistent with the findings of Cao et al.^28^. In addition, APX, GR, DHAR, and DHARM are also key components of the AsA-GSH cycle and constitute the major cellular antioxidant defense system in plants. In this study, *P. forbesii* enhanced the activities of key enzymes of the AsA-GSH cycle when subjected to HM stress, improving the stability of the AsA-GSH cycle and enhancing the antioxidant capacity of the plant. Thus, the stress resistance of *P. forbesii* can be improved by stabilizing and ensuring the normal operation of the AsA-GSH cycle. Research has shown that MT and its metabolites can chelate toxic metals in vitro^76^. For example, 24-EBR can reduce the inhibition effect of stress and promote plant growth by increasing photosynthetic pigments and AsA-GSH^77^. Additionally, 24-EBR enhances the circulation of AsA-GSH under Zn stress, thereby reducing ROS accumulation and alleviating lipid membrane peroxidation^78^, consistent with the results of this study. The application of mitigating substances elevated APX, GR, DHAR, and DHARM levels, suggesting that the application of exogenous substances may stimulate ROS reduction, inhibit lipid peroxidation, increase the efficiency of the AsA-GSH cycle, and improve the functions of enzymatic and non-enzymatic antioxidants under different stresses^79^.

### Effects of 24-EBR and MT on Ni and Cu concentrations

**Figure 5:**
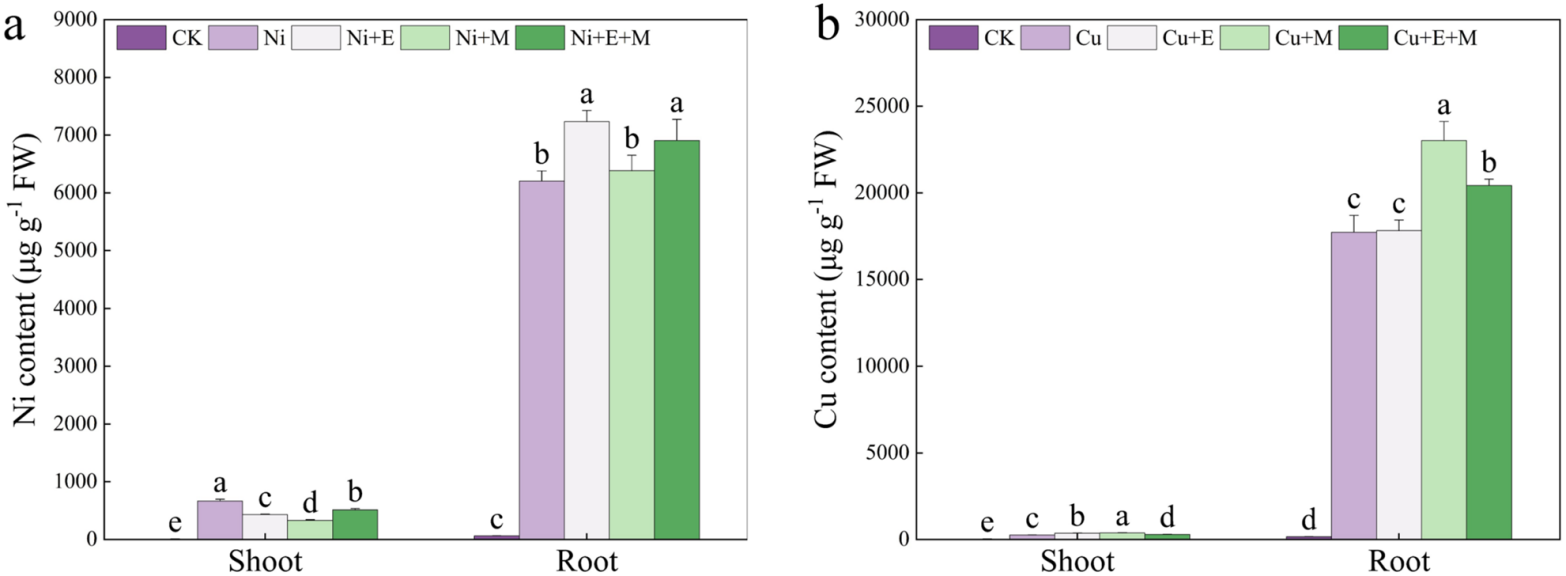
The Ni and Cu contents in the shoots and roots of *Primula forbesii* under different treatments. Data are expressed as means ± S.D. Values labeled with different lowercase letters are significantly different (*p* < 0.05) according to Duncan’s multiple range test.

Nickel stress alone significantly promoted the uptake and accumulation of Ni in *P. forbesii*. However, the mitigating substances significantly reduced Ni accumulation in the aboveground part of *P. forbesii* plants, with MT showing the greatest reduction. The mitigating substances also promoted Ni accumulation in the belowground parts of *P. forbesii*, and 24-EBR had the strongest promotional effect by causing 16.57% higher Ni accumulation than under Ni stress alone. Copper stress alone significantly increased Cu accumulation in the aboveground and especially the belowground parts of *P*. *forbesii*. However, the mitigating substances significantly increased Cu accumulation in the aboveground and belowground parts of *P. forbesii*, with significant differences between the different mitigation treatment groups. Specifically, MT treatment significantly increased Cu accumulation in both aboveground and belowground parts of *P. forbesii* relative to plants under Cu stress alone, especially in the belowground part of *P. forbesii*. Meanwhile, 24-EBR significantly promoted Cu accumulation in the aboveground part of *P. forbesii*. Additionally, MT + 24-EBR significantly increased Cu accumulation in both aboveground and belowground parts of *P. forbesii*. In general, MT significantly increased Cu accumulation in *P. forbesii* under Cu stress (Fig. 5).

Heavy metal stress increased the accumulation of HM in *P. forbesii*., mainly in the belowground part. Exogenous mitigating substances significantly enhanced the accumulation of HMs in *P. forbesii* and decreased Ni accumulation in the aboveground part of Ni-stressed *P. forbesii*, contrary to the results of Shah et al.^80^ and Jia et al.^32^. This study hypothesized that this discrepancy may stem from the ability of the exogenous substances to enhance the adsorption and accumulation of HMs in plants. In addition, the promotion of HM accumulation did not adversely affect the physiological and biochemical indexes of plants. Therefore, MT and 24-EBR enhanced resistance to HMs and the accumulation capacity of HMs in *P*. *forbesii*, potentially improving the resistance and repair capacity of the plant.

## Conclusions

Studies have shown that HM stress inhibits plant growth, blocks photosynthesis, accumulates large quantities of ROS, and increases lipid membrane peroxidation. In contrast, external hormone application can restore plant growth characteristics, enhance photosynthesis capacity, increase antioxidant enzyme activity, detoxify excess ROS, reduce oxidative damage, and enhance plant tolerance to stress. *P. forbesii* increased its HM accumulation under HM stress. Application of MT (100 μmol L^–1^) had the best mitigating effect on *P. forbesii* under Ni (200 μmol L^–1^) stress. Moreover, MT treatment enhanced the growth of *P. forbesii* under HM stress than the control. Additionally, MT improved the activities of antioxidant enzymes, and based on indicators of lipid peroxidation, MT treatment reduced MDA activity by 52.17% and superoxide anion activity by 38.80%. Besides, MT substantially increased the GR and DHAR activities of the AsA-GSH cycle, improved the cycling efficiency of AsA-GSH, and enhanced the scavenging capacity of ROS. The combined treatment of 24-EBR + MT best mitigated Cu (200 μmol L^–1^) stress. The combined treatment of 24-EBR + MT significantly enhanced the growth indexes and significantly increased the activities of antioxidant enzymes by inducing the maximum total antioxidant enzyme activity, 1.73-fold compared with the control. Additionally, 24-EBR + MT treatment significantly reduced the level of membrane lipid peroxidation and accelerated the efficiency of ASA-GSH cycling, increasing the activities of APX and MDHAR by 1.65- and 1.00-fold, respectively, compared with the control. Thus, 24-EBR and MT critically alleviate oxidative damage and reduce oxidative stress in plants. Both substances are potentially useful as mitigating substances to improve HM resistance and reduce HM-associated oxidative damage.

The threat of soil HMs to plants, the agricultural environment, and human health remains a major problem. The huge influence of these HMs destroys the root structural system of plants and hinders growth. Some progress has been made in the study of 24-EBR and MT. However, the hormone-regulated pathways under HM stress remain unclear, and future studies should further explore their molecular mechanisms.

### Ethical approval

The plants and seeds used in this article were bred by PhD. Yin Jia of the College of Landscape Architecture, Sichuan Agricultural University, and have independent intellectual property rights. All plant experiments were conducted in accordance with relevant institutional, national, and international guidelines and legislation.

## Data Availability

The datasets used and/or analyzed during the current study are available from the corresponding author on reasonable request.
